# The biomechanical role of overall-shape transformation in a primitive multicellular organism: A case study of dimorphism in the filamentous cyanobacterium *Arthrospira platensis*

**DOI:** 10.1371/journal.pone.0196383

**Published:** 2018-05-10

**Authors:** Atitheb Chaiyasitdhi, Wirat Miphonpanyatawichok, Mathis Oliver Riehle, Rungrueang Phatthanakun, Werasak Surareungchai, Worasom Kundhikanjana, Panwong Kuntanawat

**Affiliations:** 1 Biological Engineering Program, Faculty of Engineering, King Mongkut's University of Technology Thonburi, Bangkok, Thailand; 2 Division of Biotechnology, School of Bioresources and Technology, King Mongkut's University of Technology Thonburi, Bangkok, Thailand; 3 Centre for Cell Engineering, Institute of Molecular, Cell and Systems Biology, College of Medical, Veterinary and Life Sciences, University of Glasgow, Glasgow, United Kingdom; 4 The Synchrotron Light Research Institute, Nakhon Ratchasima, Thailand; 5 Nanoscience & Nanotechnology Graduate Program, Faculty of Science, King Mongkut's University of Technology Thonburi, Bangkok, Thailand; 6 School of Physics, Institute of Science, Suranaree University of Technology, Nakhon Ratchasima, Thailand; 7 School of Biotechnology, Institute of Agricultural Technology, Suranaree University of Technology, Nakhon Ratchasima, Thailand; LAAS-CNRS, FRANCE

## Abstract

Morphological transformations in primitive organisms have long been observed; however, its biomechanical roles are largely unexplored. In this study, we investigate the structural advantages of dimorphism in *Arthrospira platensis*, a filamentous multicellular cyanobacterium. We report that helical trichomes, the default shape, have a higher persistence length (*L*_*p*_), indicating a higher resistance to bending or a large value of flexural rigidity (*k*_*f*_), the product of the local cell stiffness (*E*) and the moment of inertia of the trichomes’ cross-section (*I*). Through Atomic Force Microscopy (AFM), we determined that the *E* of straight and helical trichomes were the same. In contrast, our computational model shows that *I* is greatly dependent on helical radii, implying that trichome morphology is the major contributor to *k*_*f*_ variation. According to our estimation, increasing the helical radii alone can increase *k*_*f*_ by 2 orders of magnitude. We also observe that straight trichomes have improved gliding ability, due to its structure and lower *k*_*f*_. Our study shows that dimorphism provides mechanical adjustability to the organism and may allow it to thrive in different environmental conditions. The higher *k*_*f*_ provides helical trichomes a better nutrient uptake through advection in aquatic environments. On the other hand, the lower *k*_*f*_ improves the gliding ability of straight trichomes in aquatic environments, enabling it to chemotactically relocate to more favorable territories when it encounters certain environmental stresses. When more optimal conditions are encountered, straight trichomes can revert to their original helical form. Our study is one of the first to highlight the biomechanical role of an overall-shape transformation in cyanobacteria.

## Introduction

The shape of a living organism is a result of its functional adaptation. Driven by certain selective pressures such as nutrient acquisition, cell division and predation, an adapted shape equips an organism with specific utilities [1]. This can be seen clearly in the case of the helix. It is well known that having a helical shape can help bacterial species with dispersal in their aquatic/mucosal habitats [1–3]. In *Campylobacter jejuni*, a helical shape gives the bacterium increased motility in the viscous intestinal mucus of its host and therefore the ability to cause disease [4]. Although morphological adaptions of primitive organisms are generally discussed, an overall-shape transformation, which in some case causes a nearly permanent loss of an adapted morphology, is minimally mentioned, especially in the context of biomechanical roles.

*Arthrospira*, also known by its commercial name Spirulina, is one of the best known planktonic microorganisms produced as a dietary supplement [5]. It is named (‘spira’ lat. spiral) for its recognizable helical shape. Unlike some other spiral-shaped bacteria which are single cellular, this helical filamentous cyanobacterium is multicellular, made up of cells connecting to each other in a single row [6,7]. *Arthrospira* undergoes cell division to elongate but multiplies through fragmentation [8].

In addition to its commercial potential, *Arthrospira*’s helical shape and its transformation have also attracted scientific interest for decades. The lack of specialized cells, such as akinetes and heterocysts, found in other multicellular cyanobacteria, means that *Arthrospira* has to rely purely on the alteration of its non-specialized vegetative cells to respond to the fluctating environement [7,9]. This can be seen through the overall-shape transformation of the trichome.

In general, *Arthrospira*’s default shape is helical, which enables a screw-like movement through fluids [1]. However, helical transformations in response to specific stimuli have also been reported. Exposure to highly intense photosynthetic active radiation was found to tighten the helical trichomes [10]. The response is thought to create self-shedding so that the bacterium is protected from photodamage. Helix relaxation occurs once the intensity of the photosynthetic active radiation decreases. Grazing is another such strong stimulus. The bacterium changes its helical pitch and helical handedness in response to the presence of a predator, to prevent ingestion by specific ciliate predators [1,11]. In addition, the alteration of helical handedness can also be promoted by abiotic stimuli such as a rise in temperature or mechanical stress [11].

Among all the possible transformations, linearization of the trichome is the most frequently mentioned. The straight variant has lowered metabolic rates and grows more slowly compared to the helical variant [12,13]. These two variants also differ in gene expression profiles and biochemical compositions [13].

Straight trichomes can be found spontaneously occurring in laboratory and outdoor cultures [14,15]. The ratio of helical to straight trichomes in mixed populations varies with culture condition [15]. We have observed the coexistence of straight and helical trichomes in multiple sample collecting sites (unpublished). A number of other reports have pointed out that environmental stresses can trigger a linearization transformation [12,14,16,17] and increase the population of straight trichomes [18]. For example, nutrient shortage can lead to a complete or nearly complete elimination of helical trichomes and domination of emerging straight trichomes in the population [14,15,19].

Unlike tightening or relaxation of the helix, or helical handedness alteration, which are reversible depending on the stimuli [10,11,20], linearization is highly stable and can persist even after the nutrient shortage has passed [12]. Linearization is caused by genetic variation, highly inheritable [12], and once believed to be irreversible [15]. However, a more recent study has reported that the re-emergence of helical trichomes, with the original genetic traits, in axenic cultures of straight trichomes is possible although extremely unlikely [12]. For instance, in one observation, it took 9 years for the helical trichome to emerge after the straight trichomes culture was established (bacterium doubling time ~ 24 hrs).

Previous discussions on the linearization transformation have focused on cellular physiological alterations. However, no previous studies have discussed the function of the straight structure, and the advantages gained which make it worthwhile to relinquish the adaptability of the helical structure. We hypothesize that each structure may have its own mechanical advantages under different conditions.

One of the fundamental mechanical properties for a rod-like planktonic microbe is flexural rigidity (*k*_*f*_), the resistance to bending of the structure due to exerted mechanical stress. In this study, we first estimated *k*_*f*_ of helical and straight *Arthrospira* trichomes through the calculation of persistence length (*L*_*p*_). Since *k*_*f*_ is a product of the local cell elasticity (*E*) and the moment of inertia (*I*), or a measure of stiffness at the morphological level, we investigated whether the difference in *k*_*f*_ of the trichomes was due to *E*, or *I*, or both. We performed force spectroscopy measurements with an atomic force microscope (AFM) to determine *E* of *Arthrospira* cells and then estimated *I* from geometric models of *Arthrospira* trichomes with varying radius of the helix (*R*_*helix*_). This allows us to calculate the *k*_*f*_ of the trichomes, and estimate how much the degree of coiling contributes to the stiffness. In the third part, we conducted a systematic study of the gliding motility, a form of bacterial locomotion on solid substrata, of these two morphologies to understand the impact of linearization on gliding mechanics.

## Materials and methods

### Strains, media, growth conditions

**Fig 1:**
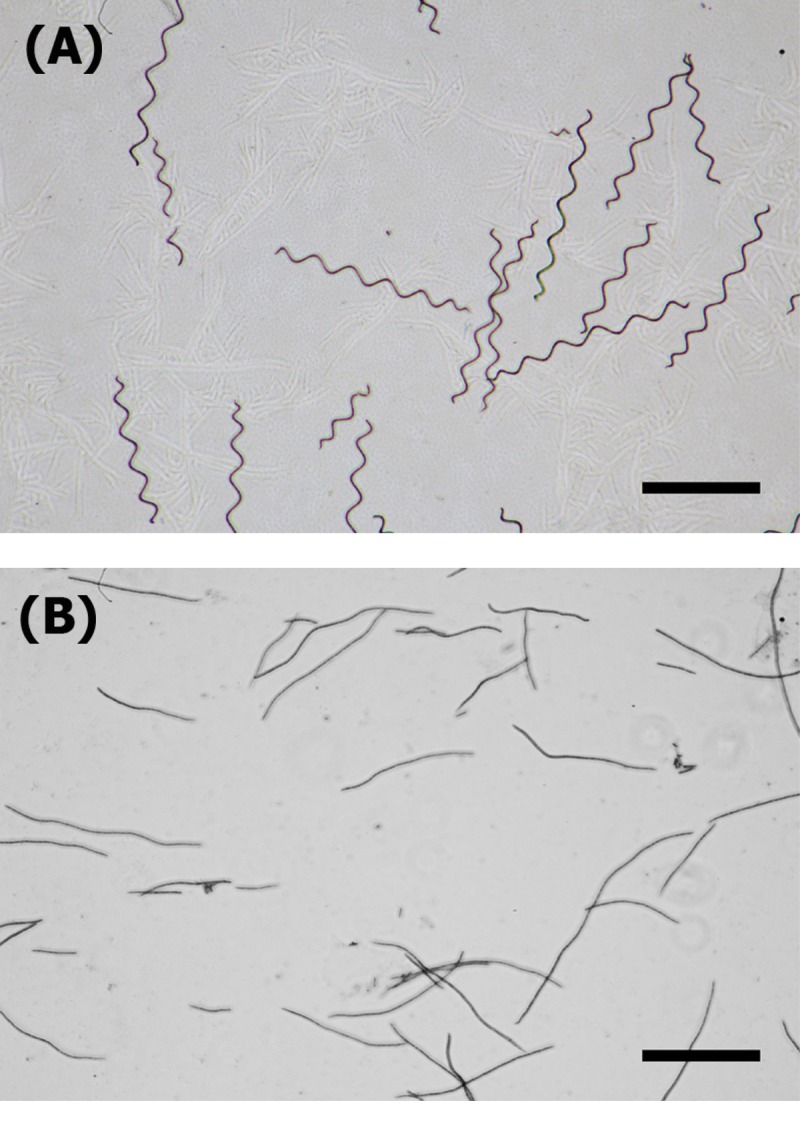
*Arthrospira platensis* strain C005. **(A)** The morphology of helical and **(B)** straight trichomes of *Arthrospira platensis* used in the experiment. Scale bars represent a length of 300 μm.

The samples used in this study were helical and straight trichomes of *Arthrospira platensis* strain C005 from the Applied Algal Research Laboratory, Chiang Mai University, (**Fig 1A and 1B**, respectively). The *Arthrospira* strain C005 originally has a helical shape. However, after several months of maintaining the strain C005 in Zarrouk's medium [21] at 30°C, illuminating with a fluorescent lamp at 60 μmol photons m^-2^s^-1^ and continuous shaking at 120 rpm, straight trichomes were found to emerge in the culture similar to previous reports in refs [12,13,15,19]. The emerging straight trichomes were then isolated and kept under the same growth conditions as the original C005 strain. These morphologically distinct variants were renamed C005-L for straight trichomes and C005-H for helical trichomes. *Arthrospira* trichomes used in this study were collected from log-phases (*OD*_*560*_ ≈ 1.2 ± 0.1).

### Estimating comparative bending resistance of *Arthrospira* trichomes from persistence lengths

In simple words, persistence length (*L*_*p*_) is a degree of bendiness—the degree of fluctuation of local orientation along the length of an object resulting from natural deformation. It is proportional to the structure’s flexural rigidity divided by the Boltzmann constant multiplied by the absolute temperature of the system (*L*_*p =*_ *k*_*f*_ */K*_*b*_*T*). We use *L*_*p*_ as a comparative indicator of resistance to bending of the trichomes. *L*_*p*_ is a parameter that can be simply obtained from optical inspection, and thus suitable as a comparative indicator of resistance to bending of the trichomes. For more theoretical background please see ref [22,23].

The procedure to measure *L*_*p*_ of *Arthrospira* trichomes is explained in our previous work [24]. In brief, first the *Arthrospira* suspension is placed in the space between a microscope slide and a coverslip with the distance between the coverslip and the microscope slide defined by two spacers (ca. 150 μm thick). This height is sufficient for the trichomes to be fixed in position but not deformed, allowing the trichome length to be viewed from the top.

The images of *Arthrospira* trichomes were taken with a 4x objective on a microscope (Eclipse Ti2, Nikon, Japan) equipped with a DSLR camera (Canon EOS 60D, Canon, Japan). Images with a resolution of 5,184x3,456 pixels were produced. Each pixel is equivalent to an actual length of about 0.47 μm. Our custom MATLAB scripts (MathWorks, USA) [24] were used to transform the light micrographs into binary images before determining the positions of the trichomes’ mid-line in Cartesian coordinations. The script then computed *L*_*p*_ according to the following definition of the tangent correlation function (*C*_*t*_*(dS)*) as a function of distance (*dS*) along the mid-line of the trichomes (Eq (1)).

$$C_{t}\left(dS\right)=exp\left(-dS/L_{p}\right)$$

The distances (*dS*) used in the calculation are in quantas of 0.47 μm, which is the smallest discrete distance in the imaging system.

As *L*_*p*_ is proportional to flexural rigidity (*k*_*f*_), which is the product of the elastic modulus (*E*) and the moment of inertia of the cross-section (*I*),

$$L_{p}=\;k_{f}/k_{B}T\;=\;EI/k_{B}T$$

we further determined whether one or both of these quantities contribute to the difference in bending resistance of *Arthrospira* trichomes in the following experiments.

### Calculating the local elasticity of *Arthrospira* cells with AFM force spectroscopy measurement

To measure the local elasticity, or elastic modulus (*E*), of *Arthrospira* cells in straight and helical trichomes, we performed force spectroscopy measurement with an AFM. Prior to the experiments, we modified a glass slide to immobilize *Arthrospira* trichomes. We built a chamber on a glass slide, incubated the structure with 0.01% Poly-L-Lysine (Sigma-Aldrich, USA) for 24 h, removed the Poly-L-Lysine and stored the dried slide in the dark for later use.

0.5 mL log phase cultures were diluted with an equal volume of de-ionized (DI) water and centrifuged at 4,300 g to pellet the cells. The cell pellet was placed into a 1.5 ml micro-centrifuge tube and washed 3 times by re-suspending the cells in 0.5 mL of DI water followed by centrifugation. Then, the re-suspended *Arthrospira* were dropped onto the Poly-L-Lysine coated glass slides, left undisturbed briefly to promote adhesion, and immediately filled with Zarrouk’s medium [21] before the experiments.

For AFM force spectroscopy measurements, we employed XEI-120 (Park System, South Korea). Measurements of both straight and helical trichomes were performed in medium using the MLCT-F tip (Bruker, USA) with a nominal resonance frequency of 125 kHz and a spring constant of 60 N·m^-1^. Topography images of trichomes were obtained to locate the mid-lines of trichomes before performing the force spectroscopy, which is achieved by pressing the AFM tip on the mid-lines of trichomes and measuring the force on the cantilever as a function of tip-sample distance. The mid-lines were selected because we found that collecting force-distance curves from positions not perpendicular to the AFM tip increased the probability of measurement artifacts due to a high aspect ratio of the lateral sides (~ 8–10 μm height) compared to the tip. To calculate *E*, we fit a force-distance curve to the modified Hertz model for pyramidal tips as referenced by [25,26]. MATLAB scripts written by the authors combined with custom MATLAB scripts from [26] were used in this stage.

### Estimating moment of inertia of the cross-section flexural rigidity by using geometric modeling

Estimating the moment of inertia of the cross-section (*I*) of *Arthrospira* trichomes requires constructing a model of trichomes from their structural parameters. All morphological parameters, except the inner wall thickness, were measured using the Fiji image analysis software [27]. The trichomes were modelled as isotropic hollow cylindrical rods with thin walls and blunt ends coiled into a helical shape with radii (*R*_*helix*_) varying from *R*_*helix*_ = 0 μm (to represent a straight trichome) to 15 μm (to represent a helical trichome). The inner wall thickness was equal to 90 nm as reported in [7]. The radius of trichomes (*R*_*trichome*_) was 5 μm and the contour length, or total length from one end to the other, was 300 μm. An assumption of isotropic hollow cylindrical tubes has previously been proven to work well in the modeling of multicellular filamentous structure [28]. The elastic map of helical and straight *Arthrospira* obtained using AFM force spectroscopy revealed that the local cell elastic moduli appeared uniform across the trichomes of both morphologies (see supplementary **S1 Fig**), further validating the modeling of the trichomes as isotropic rods.

*I* was computed with the finite element method according to the definition in Eq (3) [23].

$$I=\;\int_{cross-section}^{}y^{2}dA$$

We assumed that the bending moment occurs in the direction perpendicular to the x-axis; therefore, *I* relative to the neutral axis x (*I*_*xx*_) can be estimated from the cross-sectional area. The integration is performed over the cross-section of the hollow rods by using custom MATLAB scripts. In our model, the cross-sectional area at the middle (*L* = 150 μm) of the model trichomes with varying radii of helix were used to estimate *I*_*xx*_. We also estimated *k*_*f*_ of the model trichomes by using the elastic modulus measured from the AFM force spectroscopy.

### Gliding motility of helical and straight *Arthospira* trichomes

All the gliding experiments were conducted using a chemically uniform Zarrouk’s agar (Zarrouk’s medium with 1.8% agarose added) under uniform background lighting. No known external stimuli were included to bias bacterial movement. 10 ml hot sterile Zarrouk’s agar was poured into a 4” petri dish and left to set in a thin flat layer. 20 μl of ~10,000 trichomes/ml *Arthrospira* suspension containing helical, straight or a mixture of both types, was carefully dropped in the center of the agar. The petri dish was placed on the microscope before proceeding with the imaging. The computerized time-lapse imaging of the trichomes moving within a static field of view was done at a frequency of 5 minutes/frame for up to 600 minutes using EOS utility (Canon, Japan). The microscope and imaging system were similar to that used in capturing the still images of *Arthrospira* previously described.

Each sequence of images was combined into a stack and further analyzed using Fiji. The individual trichome’s gliding trajectory within the field was tracked using the plugin Manual Tracking provided in the software. The coordinate at each step of each trajectory was collected and used to calculate two primary quantities: the length of gliding trajectory (*D*) and the Euclidian length of the gliding trajectory (*d*)—a straight line from the start to the end point of the gliding path. After this, the speed over each individual gliding event (*v; D*/duration of the gliding) was calculated. The directionality ratio (*d/D*) was also computed to quantify the straightness of the gliding. A directionality ratio closer to 1 indicates straighter or more directional motility [29].

## Results

### Persistence length as an indicator of bending resistance of the trichomes

**Fig 2:**
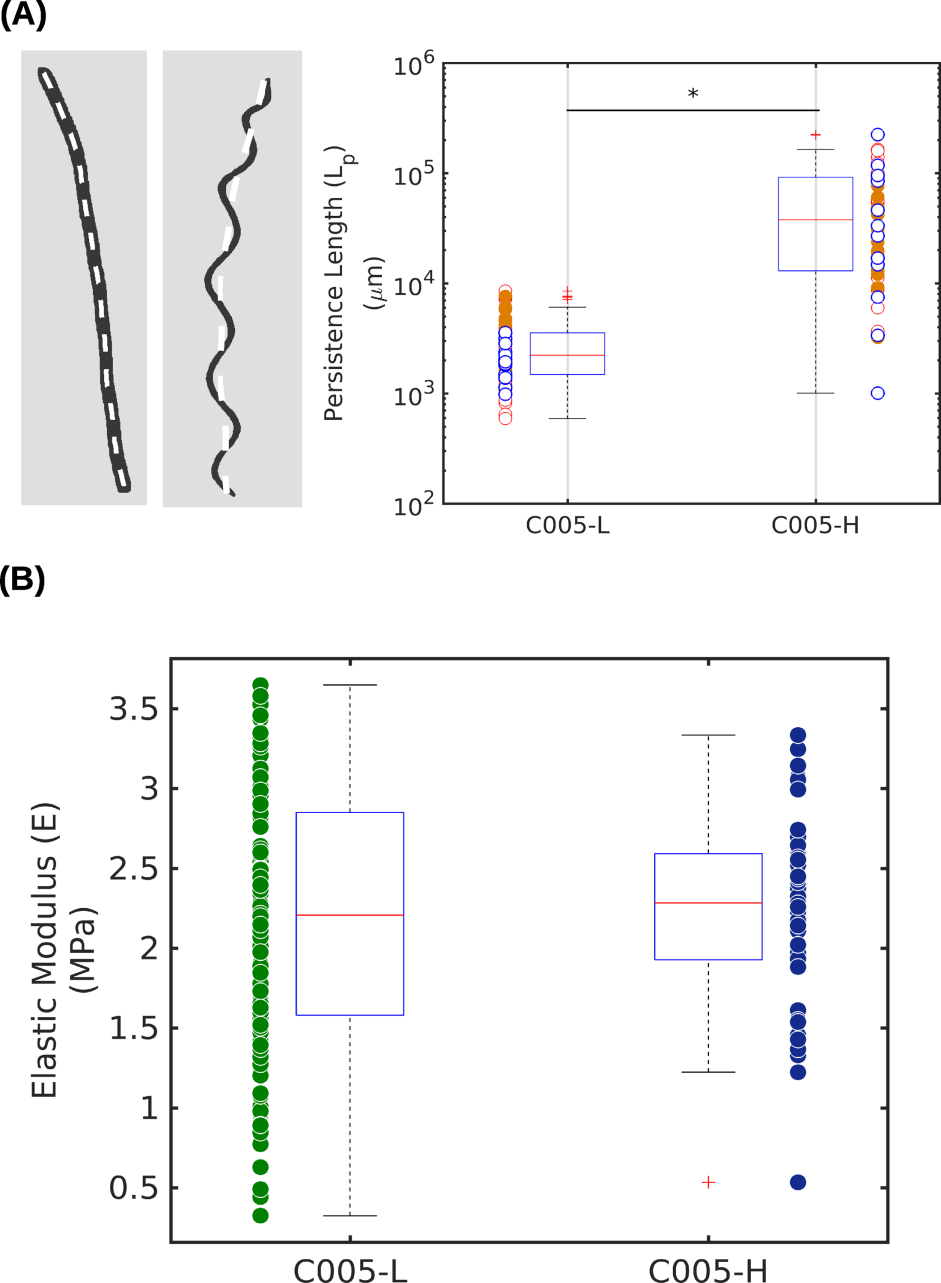
The mechanical properties of *Arthrospira* trichomes. **(A)** The persistence length (*L*_*p*_) of straight (*Median* ± *SD* = [2 ± 3] x 10^3^ μm) and helical trichomes (*Median* ± *SD* = [37 ± 58] x 10^3^ μm) measured along the center of the trichomes represented by white dash lines (left panel). Helical trichomes have a statistically significant greater *L*_*p*_ than straight trichomes (t-test, *p* < 0.001). The values shown in **(A)** are raw data from 3 replicates and represented by boxplots. The circles in the boxplots represent the medians from each replicate. **(B)** Elastic moduli (*E)* calculated from the middle of straight (*Median* ± *SD* = 2.3 ± 0.5 MPa) and helical trichomes (*Median* ± *SD* = 2.4 ± 0.5 MPa) have no significant difference (t-test, *p* = 0.722, α = 0.01). Raw data is represented by boxplots with the same manner as in **(A)**. All statistical testing in this section was performed with the MATLAB Statistical Package.

To measure *L*_*p*_ of *Arthrospira* trichomes, we captured images of the trichomes under a light microscope and calculated *L*_*p*_ by using custom MATLAB scripts. The calculations revealed that helical trichomes had significantly greater *L*_*p*_ than straight trichomes, with a significant difference at α = 0.01 (t-test, *p* < 0.001). The median of *L*_*p*_ for straight and helical trichomes were [2 ± 3] x 10^3^ μm (± *SD*) and [37± 58] x 10^3^ μm (± *SD*) respectively **(Fig 2A)**. In addition, the *L*_*p*_ of the straight *Arthrospira* trichomes measured in our experiment were close to that of other *Oscillatoria* trichomes reported by Boal and Ng [22]. Therefore, the data indicates that helical trichomes are stiffer than straight trichomes according to Eq (2). The *L*_*p*_s are much longer than the actual length of these two planktons mean that they are ‘stiff ‘ in the static suspension, but still deformable under the background shear flow as demonstrated in previous studies on diatom chains [30,31].

Since both elastic modulus *(E)* and the moment of inertia *(I)* contribute to *L*_*p*_ as shown in Eq (2), we measured *E* and *I* in the following sections to identify the major contributors to the higher *k*_*f*_ of helical trichomes.

### Elastic moduli of *Arthrospira* cells measured by AFM force spectroscopy

A higher elastic modulus (*E*) of cells in one of the two *Arthrospira* variants would indicate higher resistance to bending. To calculate *E* of *Arthrospira* cells, we performed AFM force spectroscopy along the middle of the trichomes to obtain force-distance curves and calculated *E* by using custom MATLAB scripts.

For straight and helical trichomes, *E* was 2.3 ± 0.5 MPa and 2.4 ± 0.5 MPa respectively (**Fig 2B**). These values sit between that of mammalian cells (0.1–400 kPa) [32] and cyanobacterium *Nostoc* (20 ± 3 MPa) [33].

The results suggest no significant difference at α = 0.01 (t-test, *p* = 0.722). This finding is consistent with the study by Hongsthonget *et al*. which showed that carbohydrates in straight and helical trichome cells are the same [13]. Similar *E* values between the two trichome morphologies imply that the moment of inertia (*I*) is likely the major contributor to the higher resistance to bending in helical trichomes.

### Estimation of moment of inertia and flexural rigidity from geometric modeling

The moment of inertia of the cross-section (*I*) is defined as the area-weighted integral of the square distance from an axis [23,34] and represents the contribution of morphology to stiffness at the morphological level. To estimate *I* for straight and helical trichomes, we generated models of *Arthrospira* trichomes with varying radii of the helix (*R*_*helix*_) based on their structural parameters. The *E*s used in the models were obtained from the AFM experiment and finally both *I* and *E* were used to calculate flexural rigidity (*k*_*f*_), as a more general measure for bending resistance.

**Fig 3:**
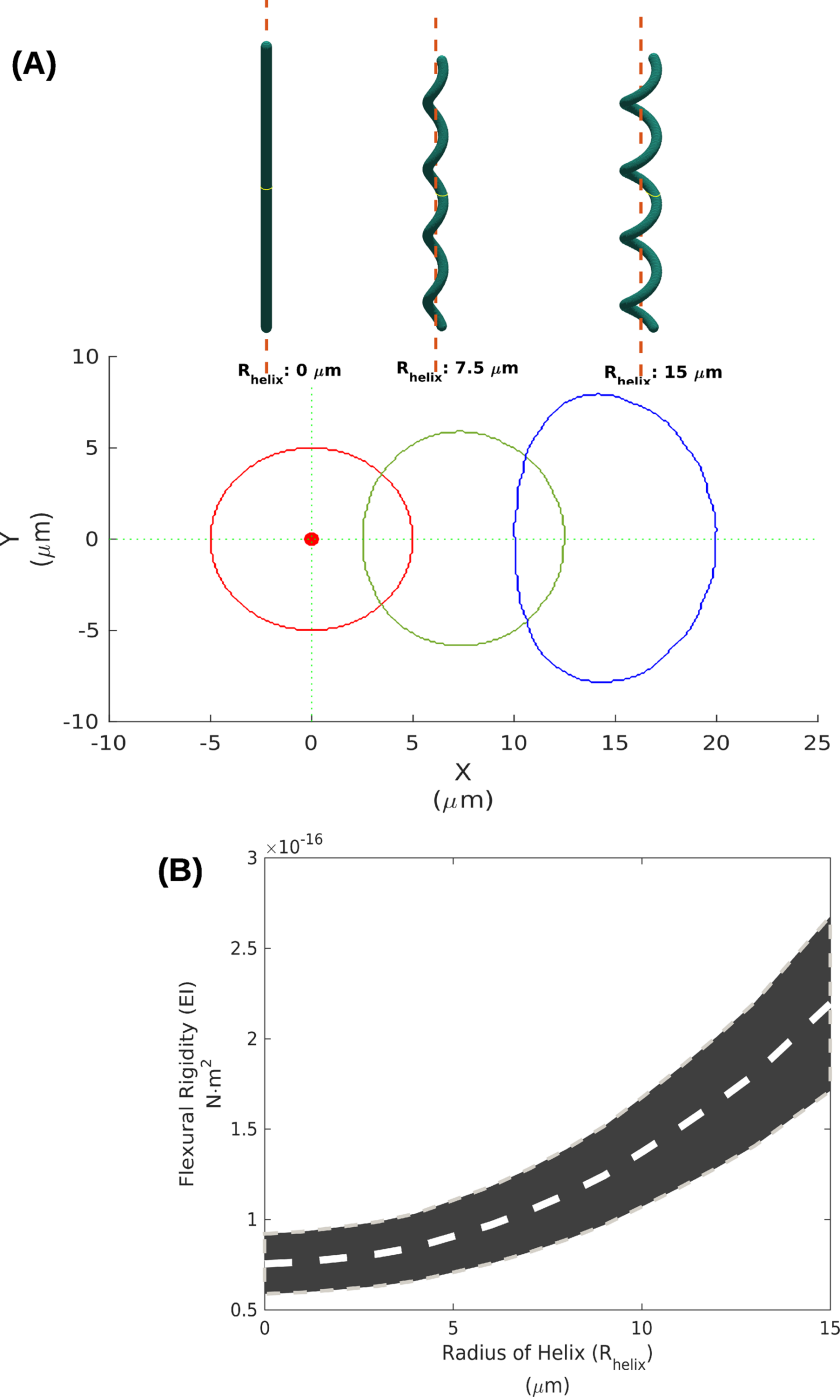
Estimating flexural rigidity. **(A)** The 3D geometric models of a straight trichome with *R*_*helix*_ = 0 μm, and helical trichomes with *R*_*helix*_ = 7.5 and 15 μm were constructed as thin rods (upper panel). The cross-sections from the middle of trichomes (*L* = 150 μm) moves away from the centroid as *R*_*helix*_ increases (lower panel). **(B)** The estimated flexural rigidity (*k*_*f*_) increases as *R*_*helix*_ increases. The gray stripe indicates the possible range of *k*_*f*_ calculated from the measured *E*.

We found that as *R*_*helix*_ increased, the cross-section area of the trichome expanded and moved away from the centroid (**Fig 3A)**, thus increasing the estimated *k*_*f*_ from 0.07x10^-16^ N·m^2^ for *R*_*helix*_ = 0 μm (straight) to 2.19x10^-16^ N·m^2^ for *R*_*helix*_ = 15 μm (helical trichomes) **(Fig 3B)**. these estimated *k*_*f*_ values are in the same range as that of diatoms obtained from the aspiration method as reported in [30]. The increase of the estimated *k*_*f*_ follows the definition of *I* that *k*_*f*_ can increase with a power of four to the radius of mass away from the centroid. The results showed that coiling of *Arthrospira* trichomes alone can greatly increase *k*_*f*_, by 2 orders of magnitude, resulting in higher bending resistance for helical trichomes than straight trichomes.

### Gliding motility of helical and straight *Arthrospira* trichomes

**Fig 4:**
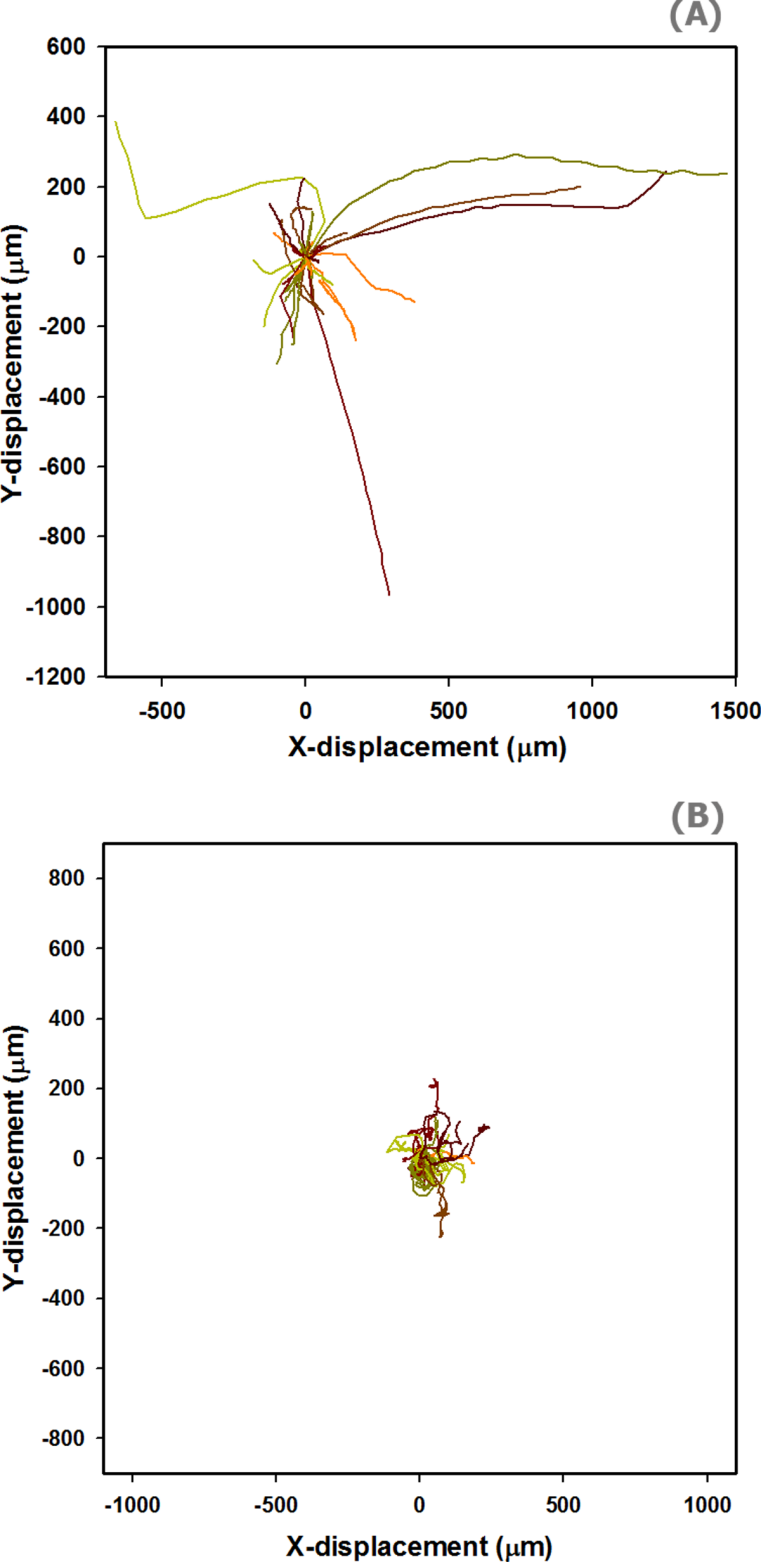
Patterns of gliding trajectories of helical and straight trichomes. Examples of the trajectories here were constructed from the position of the gliding trichomes on the solid media’s surface within the static field of view collected every 5 minutes. The starting point of the trajectories presented in these figures is set at the origin, (0, 0), for the purpose of visualization. **(A)** Gliding patterns of the straight trichomes are more directional. The trajectories tend to point away from the starting point in particular directions. Some trichomes were found to switch directions. Snap turns were also observed occasionally. **(B)** On the other hand, gliding patterns of the helical trichomes are less directional. The trajectories involve frequent random turns and twists.

Positions of 32 straight and 51 helical trichomes gliding on the chemically uniform solid agars, collected every 5 minutes over the course of up to 600 minutes, were used in reconstructing trajectories of trichomes gliding motility. Examples of some trajectories are presented in **Fig 4**. It should be noted that some of the trichomes left the field of view before the 600 minute course of observation was completed, which automatically terminated the recording of these particular trajectories. The trajectories presented in **Fig 4**cannot therefore be directly compared in terms of distance the bacteria moves over the fixed course of time. **Fig 4**however displays the distinctive patterns between straight and helical trichomes.

**Fig 5:**
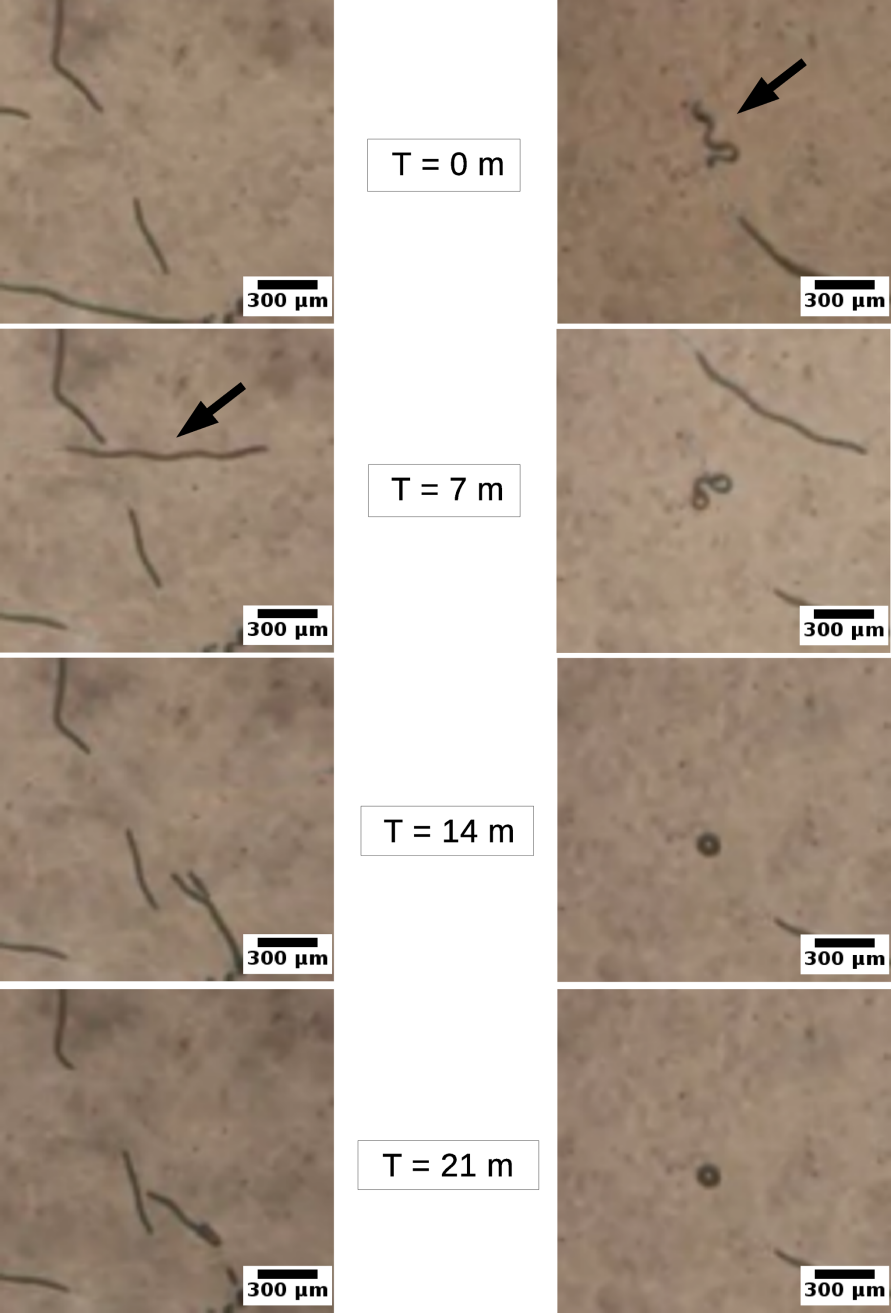
Gliding motility. Straight *Arthrospira* trichomes (left panel) exhibited better gliding motility and were able to move for a longer distance, while helical trichomes (right panel) remained near the initial positions. The image sequences of both strains were taken at *t* = 0, 7, 14 and 21 min respectively. The arrows represent the positions of trichomes (see **S1 Movie.**).

In general, linear trichomes exhibited more elongated trajectories pointing away from the starting point (**Fig 4A)**. We found that some trichomes might move back and forth occasionally. Snap turns during gliding were also observed. In contrast, helical trichomes generally failed to exhibit any directionality in their movement (**Fig 4B)**. The gliding comprises of frequent random turns. Most of the trichomes twisted around their starting point, curled and, in extreme cases, ended up back near their initial position. In addition, the helical trichomes deformed their curving structures over time as seen in the supplementary movie (see **S1 Movie**), suggesting a certain amount of the force generated from the subcellular propelling motion was wasted. The deformation also caused the position of the trichomes’ leading end to fluctuate over time, which translated into direction changes and reduced directionality. Examples of straight and helical trichome gliding can be seen in **Fig 5**and the supplementary movie (see **S1 Movie**).

**Fig 6:**
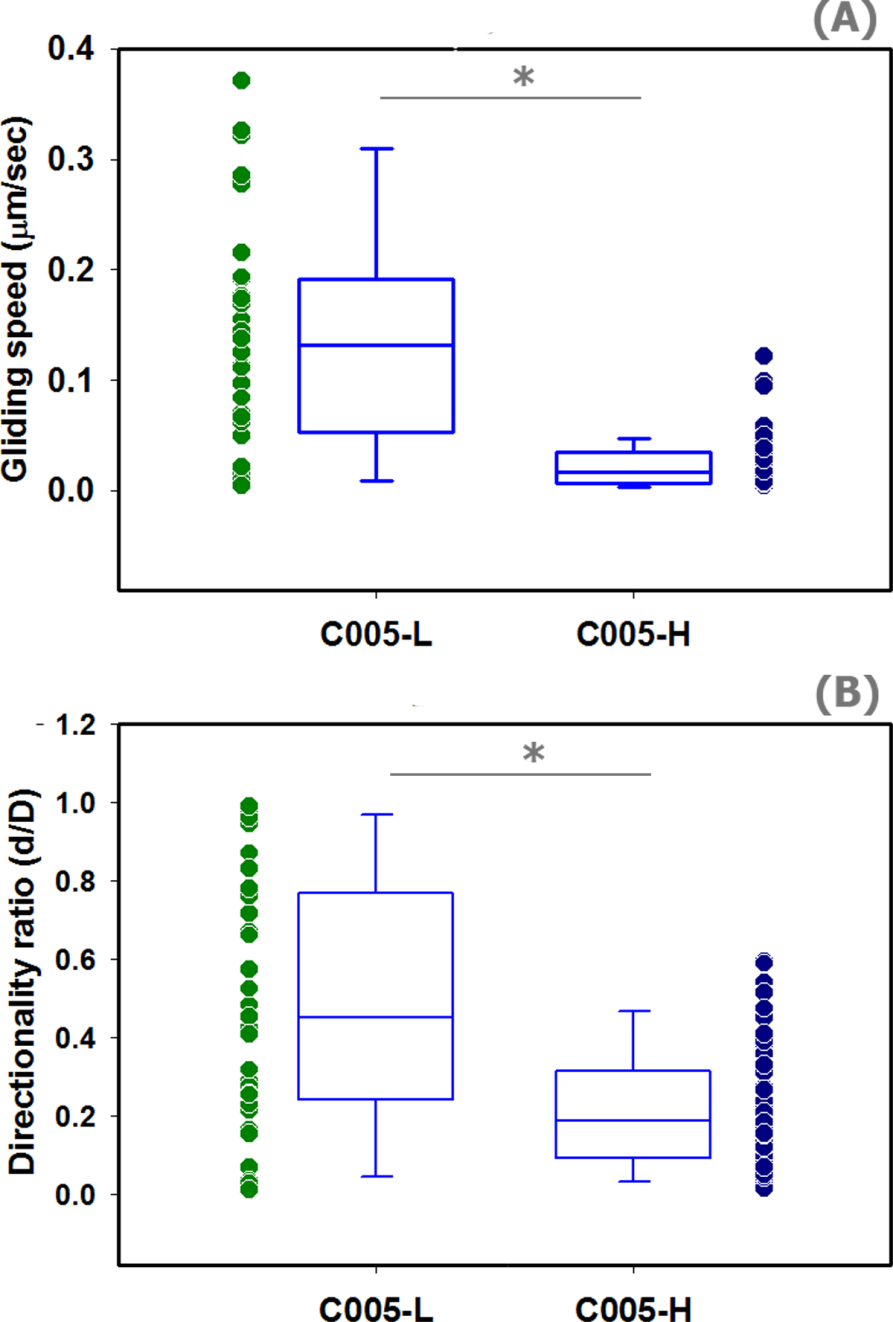
**Speed and directionality ratio of the gliding motility of straight and helical trichomes (A)** The scatter and box plots represent the raw data and distribution for the trichomes’ gliding speed (*v*) respectively. Based on data collected from 32 straight and 51 helical trichomes, the average *v* of straight and helical trichomes (*median* ± *SD*) are 0.13 ± 0.10 μm/sec and 0.02 ± 0.02 μm/sec respectively. The gliding speeds of the two morphologies are significantly different (t-test, *p* < 0.001). **(B)** The scatter and box plots represent the raw data and distribution for the trichomes’ gliding directionality ratio (*d/D*) respectively. Based on data collected from 32 straight and 51 helical trichomes, the average *d/D* of straight and helical trichomes (*median* ± *SD*) are 0.45 ± 0.32 and 0.19 ± 0.16 respectively. The gliding directionality ratios of the two morphologies are significantly different (t-test, *p* < 0.001). There is no significant correlation between *d/D* and duration of gliding in both straight and helical trichomes (Pearson Product Moment Correlation, *p* = 0.149 and 0.153, respectively, α = 0.05).

Further quantitative analysis reveals that straight trichomes have a better gliding motility in terms of speed and directionality. Gliding speed of the straight trichomes (0.13 ± 0.10 μm/sec, *median* ± *SD*) is significantly greater than that of the helical ones (0.02 ± 0.02 μm/sec, *median* ± *SD*) (t-test, *p* < 0.001), see **Fig 6A**. This indicates that on average straight trichomes can relocate more substantial distances in comparison to helical trichomes, by almost an order of magnitude. In addition, we also found that the range of the gliding speed of straight and helical *Arthrospira* (0.01–0.37 and 0.00–0.12 μm/sec respectively) lies in between that of two other cyanobacterial genera previously reported: *Synechocystis* (0.03–0.07 μm/sec) [35] and *Nostoc* (1–10 μm/sec) [36]. Here, for the first time, we demonstrate that the morphological variants of the same cyanobacterium can exhibit significantly different gliding performances.

We also calculated directionality ratio (*d/D*) which defines the straightness of the trajectories [29]. The average directionality of gliding of the straight trichomes (0.45 ± 0.32, *median* ± *SD*) was significantly greater than that of the helical trichomes (0.19 ± 0.16, *median* ± *SD*) (t-test, *p* < 0.001), see **Fig 6B**. It was noted in other literature [29] that the apparent *d/D* of cell migration may quickly decay by prolonging the period of track recording, however, this is not the case in our study. We found that there was no significant relationship between *d/D* and duration of gliding in both straight and helical trichomes (Pearson Product Moment Correlation, *p* = 0.149 and 0.153, respectively, *α* = 0.05). These results complement the previous descriptive result shown in **Fig 4**that straight trichomes have a more efficient gliding motility in terms of speed and directionality.

## Discussion and Conclusion

We demonstrated that helical trichomes have greater persistence lengths by orders of magnitude, indicating higher resistance to bending than straight trichomes. This property is also reflected in the flexural rigidity (*k*_*f*_). The lower *k*_*f*_ in the straight trichomes was solely due to the uncoiling of the trichomes, not differences in local cell elasticity. We believe that the higher *L*_*p*_ and *k*_*f*_ provides several biomechanical advantages to helical trichomes.

Similar to a diatom chain with the same order of *k*_*f*_ [30], a higher *k*_*f*_ allows *Arthrospira* to tumble more efficiently in the background shear flow, which improves access to nutrients through advection [30,37]. This may be the reason why the helical shape is the common form of *Arthrospira* in nature.

For *Arthrospira* to improve its stiffness, based on the fact that *k*_*f*_ is proportional to *E* and *I*, it can either increase the cell stiffness (*E*) or change the morphology (*I*). Through AFM nano-indentation, we confirmed that the elastic moduli (*E*) of both trichomes were the same at the cellular level. On the other hand, our modeling indicates that increasing the coiling radius is the major contributor to higher stiffness. We hypothesize that the latter is the chosen strategy because it is a relatively ‘cost effective’ (materials, time, and effort) mechanism. To increase *E*, the bacterium would have to invest substantially in intricate biosynthesis of stronger structural materials or subcellular strengthening mechanisms, such as cell wall thickening or improved peptidoglycan cross-linkage. It is not known how the helical shape forms in *Arthrospira*. However, studies in other single cell bacteria [38,39] and higher plants [40] indicate that helical shapes require only the localization of ordinary mechanically reinforcing [40,41] or structural molecules such as actin-like molecules and lignin that promote differential growth along the structure [42,43].

Although linearization limits the nutrient fluxes in aquatic habitats, it may benefit the bacterium in non-aquatic situations. Our study shows that straight trichomes have better gliding motility in terms of gliding speed and directionality on dryer solid substrate.

Gliding is a form of bacterium locomotion that has been reported for more than a century [44]. It is known to be photo- and chemotactile [35,36]. However, its underlying mechanism is inconclusive. Two major theories are that the motility is generated by cellular extrusion of polysaccharide [45] or the traction of Type IV pili [46]. Both of these highlight the fact that the subcellular gliding activity is the fundamental engine of locomotion.

The morphological variants of the same cyanobacterium exhibited significantly different gliding performances. A fundamental question is whether the increased gliding performance in straight trichomes is due to the activation of the subcellular gliding activity as a result of physiological alteration or as a result of the overall shape transformation. Although we cannot rule out physiological differences between the two shapes, the physical transformation does provide advantages for gliding. The natural wavy structures of the helical trichomes makes gliding more difficult. The deformation of helical trichomes during gliding indicates losses in propelling energy. On the other hand, straight trichomes can glide easily and can make snap turns, an action not found in helical trichomes. The lower flexural rigidity probably helps the trichomes to move through higher turning angles. This ability to turn easily would allow for more efficient exploration of a surface in the search for an optimal growth environment in their natural habitat.

The improved gliding motility may be a strategy for *Arthrospira* to preserve the population of the species when encountering certain stressful conditions, such as drought or prolonged starvation. Once triggered by the hostile environmental conditions, the straight trichomes, which have improved gliding efficiency, can chemotactically escape hostile habitats via terrestrial barriers. Nutrient flux is no longer an important consideration; thus the loss of the helical shape is an acceptable tradeoff. Because *Arthrospira* are not equipped with specialized cells, such as akinetes, that serve as survival structures in other species [9], nor sexual reproduction or lateral gene transfer that helps other species of bacteria to increase genetic diversity [7], gliding seems to be one of a few, if not the only, available adaptations *Arthrospira* can afford.

Our findings allow us to draw two possible conclusions regarding the linearization transformation: 1) *Arthrospira*’s straight shape improves gliding efficiency; and 2) this morphology can help the bacterium to migrate. The re-establishment of the helical population in different unconnected aquatic habitats can take place once a few straight trichomes revert back to the helical form with the original genetic traits.

Our finding is the first to study the advantage a multicellular primitive organism can draw from changing its overall shape. The results of this study may help us gain insights into the evolutionary history and interplay between the forms and functions of *Arthrospira* trichomes. The benefit of morphological transformations reported in this study paves the way to understanding the diversity of forms and the relation between form and function observed in other organisms, in particular those with curved and coiled structures.

## Supporting information

S1 Fig Elastic maps of a helical (above) and straight trichomes. The elastic maps were constructed from AFM force-spectroscopy measurement across the trichomes. It can be seen that the elasticity maps appear uniform along the trichomes’ length. The trichome can therefore be seen as a homogenous mechanical entity, as least in term of stiffness.
(TIF)

S1 Movie Gliding motility of *Arthrospira* trichomes. (MP4)
