# Supplementary figures and images for: The biomechanical role of overall-shape transformation in a primitive multicellular organism: A case study of dimorphism in the filamentous cyanobacterium Arthrospira platensis
Source: PLoS One. 2018 May 10;13(5):e0196383. doi: 10.1371/journal.pone.0196383 (PMC5945045; doi:10.1371/journal.pone.0196383)

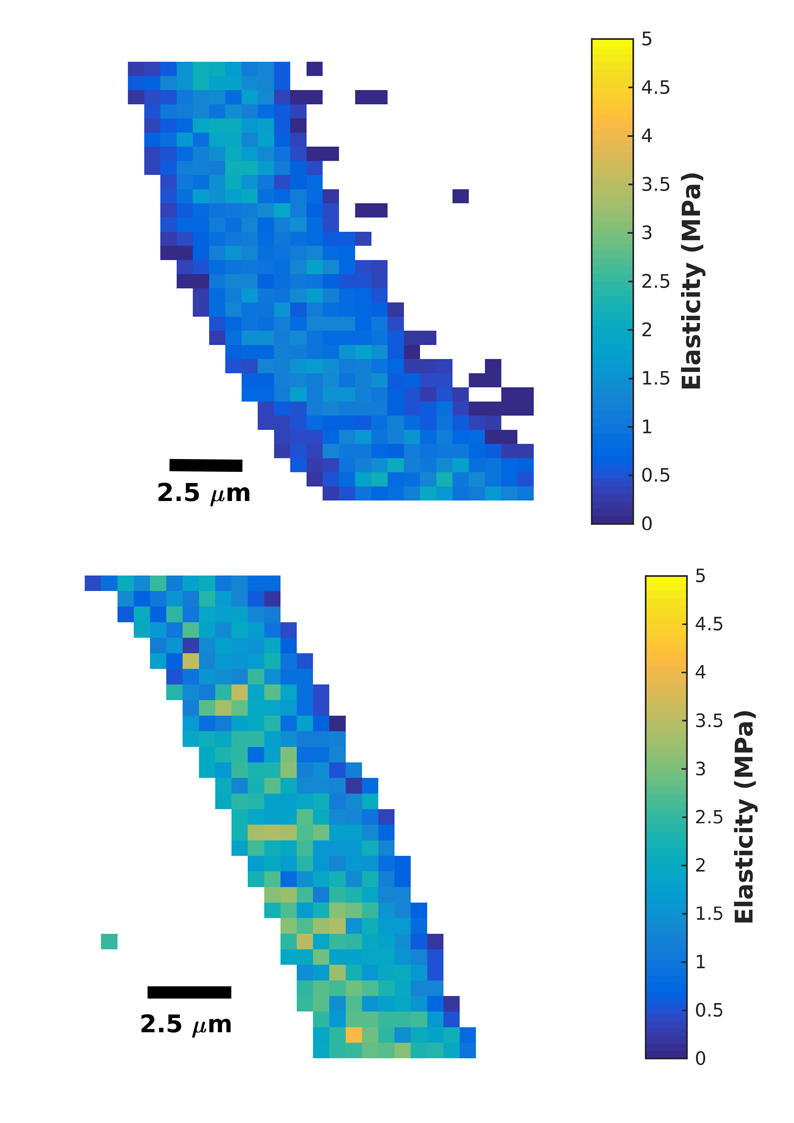

Supplement: S1 Fig — The elastic maps were constructed from AFM force-spectroscopy measurement across the trichomes. It can be seen that the elasticity maps appear uniform along the trichomes’ length. The trichome can therefore be seen as a homogenous mechanical entity, as least in term of stiffness. (TIF) [file pone.0196383.s001.tif]
